# Multifactor artificial intelligence model assists axillary lymph node surgery in breast cancer after neoadjuvant chemotherapy: multicenter retrospective cohort study

**DOI:** 10.1097/JS9.0000000000000621

**Published:** 2023-10-11

**Authors:** Teng Zhu, Yu-Hong Huang, Wei Li, Yi-Min Zhang, Ying-Yi Lin, Min-Yi Cheng, Zhi-Yong Wu, Guo-Lin Ye, Ying Lin, Kun Wang

**Affiliations:** a Department of Breast Cancer, Cancer Center, Guangdong Provincial People’s Hospital (Guangdong Academy of Medical Sciences), Southern Medical University; b Department of Breast Cancer, The First People’s Hospital of Foshan, Foshan; c Breast Disease Center, The First Affiliated Hospital, Sun Yat-sen University; d Shantou University Medical College, Shantou, Guangdong; e Diagnosis and Treatment Center of Breast Diseases, Shantou Central Hospital; f Clinical Research Centre & Breast Disease Diagnosis and Treatment Centre, Shantou Central Hospital, Shantou, People’s Republic of China

**Keywords:** axillary lymph node metastasis, breast cancer, longitudinal radiomics, machine learning, neoadjuvant chemotherapy, sentinel lymph node biopsy

## Abstract

**Background:**

The high false negative rate (FNR) associated with sentinel lymph node biopsy often leads to unnecessary axillary lymph node dissection following neoadjuvant chemotherapy (NAC) in breast cancer. The authors aimed to develop a multifactor artificial intelligence (AI) model to aid in axillary lymph node surgery.

**Materials and Methods:**

A total of 1038 patients were enrolled, comprising 234 patients in the primary cohort, 723 patients in three external validation cohorts, and 81 patients in the prospective cohort. For predicting axillary lymph node response to NAC, robust longitudinal radiomics features were extracted from pre-NAC and post-NAC magnetic resonance images. The *U* test, the least absolute shrinkage and selection operator, and the spearman analysis were used to select the most significant features. A machine learning stacking model was constructed to detect ALN metastasis after NAC. By integrating the significant predictors, we developed a multifactor AI-assisted surgery pipeline and compared its performance and false negative rate with that of sentinel lymph node biopsy alone.

**Results:**

The machine learning stacking model achieved excellent performance in detecting ALN metastasis, with an area under the curve (AUC) of 0.958 in the primary cohort, 0.881 in the external validation cohorts, and 0.882 in the prospective cohort. Furthermore, the introduction of AI-assisted surgery reduced the FNRs from 14.88 (18/121) to 4.13% (5/121) in the primary cohort, from 16.55 (49/296) to 4.05% (12/296) in the external validation cohorts, and from 13.64 (3/22) to 4.55% (1/22) in the prospective cohort. Notably, when more than two SLNs were removed, the FNRs further decreased to 2.78% (2/72) in the primary cohort, 2.38% (4/168) in the external validation cohorts, and 0% (0/15) in the prospective cohort.

**Conclusion:**

Our study highlights the potential of AI-assisted surgery as a valuable tool for evaluating ALN response to NAC, leading to a reduction in unnecessary axillary lymph node dissection procedures.

## Introduction

Highlights

- Longitudinal radiomics machine learning can accurately predict axillary lymph node metastasis for breast cancer with a negative sentinel lymph node.
- A multifactor artificial intelligence (AI) model can assist surgery and reduce the false negative rate of the current sentinel lymph node biopsy.
- The AI-assisted surgery pipeline was validated in large-sample external validation cohorts and prospective cohort.
- For patients with negative sentinel lymph node removal, an AI-assisted surgery pipeline can guide omitting axillary lymph node dissection.

Breast cancer is the most common cancer and the leading cause of cancer-related death in female worldwide. Neoadjuvant chemotherapy (NAC) is increasingly utilized in patients with clinically positive axillary lymph nodes (ALN) breast cancer. Accurately identifying the ALN status is crucial for determining the optimal axillary surgical strategy following NAC. The standard procedure for breast cancer with positive ALN after NAC is axillary lymph node dissection (ALND), but it can lead to complications such as lymphedema, arm pain, or paresthesia. NAC can eliminate axillary lymph node metastasis in 40–75% of cases with ALN+ breast cancer^1–3^. Sentinel lymph node biopsy (SLNB) is a less invasive alternative to ALND for patients who initially have ALN+ breast cancer but converted to negative after NAC. However, SLNB has a high FNR, especially when fewer than three sentinel lymph nodes (SLN) are removed^4,5^. Invasive techniques such as dual tracer staining, removal of more than three SLNs, and the use of positioning pins have improved the accuracy of SLNB, but they may not be eligible for all patients^4–7^. When all the removed SLNs were negative, it becomes challenging for the surgeon to determine the optimal next step. Thus, there is an urgent need to develop a reliable approach to accurately predict axillary lymph node response to NAC, and to predict nonsentinel lymph node (NSLN) metastasis for breast cancer with intraoperative negative SLNs.

Medical imaging-based machine learning models show great potential in accurately determining ALN status before surgery. Breast MRI is recommended for detecting tumor response to NAC and provides comprehensive information on nodal status^8^. Various parameters, such as including diffusion measures and MRI texture parameters, have been explored investigated to predict tumor response^9–14^. Radiomics features extracted from multiparametric MRI can detect tumor response to NAC in breast cancer^15,16^. However, previous radiomics models predicting ALN metastasis were developed solely based on the primary tumor since the coverage of the axilla is limited in breast MRI examination^15,17,18^. The diagnostic performance of MRI for ALN assessment has been significantly improved with the utilization of new technologies such as dedicated coils, updated contrast agent protocols, and the creation of coronal plane images^19,20^. It has been reported that standard MRI covering only the lower axillae is sufficient for excluding advanced ALN metastasis^21^. A preoperative model based on MRI radiomics, focusing on both ALN and tumor regions, was explored to predict ALN metastasis in patients with early-stage breast cancer and achieved excellent performance^22^. Additionally, most radiomics studies extracted radiomic features from a single timepoint image, which might overlook tumor and lymph node changes during NAC^15–18^. Longitudinal radiomics, which calculates the relative net changes of radiomic features, can directly reveals tumor and lymph node changes from different MRI timepoints and potentially aid in evaluating the axillary lymph node response to NAC^23^.

This multicenter study aimed to develop a novel longitudinal radiomics model based on multitimepoint and multi-region analyses on MRI images to accurately predict ALN metastasis after NAC for breast cancer. Furthermore, we explored the potential of combining the longitudinal radiomics model with other clinical and surgical factors, to assist individual clinical decision-making for patients with removed negative SLN after NAC.

## Materials and methods

### Study design

Given the retrospective nature of this study, the requirement for patient approval or written informed consent was waived. The primary cohort for this study consisted of patients from institution 1, while three other institutions provided independent external validation cohorts. Additionally, patients from two prospective clinical trials were used as the prospective validation cohort for secondary analysis. This study has been reported in line with the strengthening the reporting of cohort, cross-sectional, and case–control studies in surgery (STROCSS) criteria^24^ (Supplemental Digital Content 1, http://links.lww.com/JS9/B183).

Consecutive patients with invasive breast cancer who underwent NAC treatment and subsequent surgery between 1 January 2012 and 31 December 2021, were retrospectively enrolled. Sequential MRI scans conducted before and after NAC were acquired, along with available clinical information. The inclusion criteria were as follows: (i) biopsy-proven invasive breast cancer; (ii) patients with clinically node-positive disease who received NAC treatment; (iii) patients who underwent SLNB and ALND; (iv) pre-NAC and post-NAC available MRI images; (v) available baseline data. The exclusion criteria were as follows: (i) patients underwent SLNB before NAC; (ii) presence of occult breast tumors; (iii) unqualified MRI images (obvious artefacts, incomplete sequences, or signal strength differences caused by displacement); (iv) presence of distant metastasis or another malignancy.

### NAC and pathological assessment

NAC treatment was administered in accordance with the National Comprehensive Cancer Network (NCCN) guidelines, and all patients completed either 6 or 8 cycles of NAC. The NAC regimens were based on taxane or a combination of taxane and anthracycline. Additionally, all patients with a human epidermal growth factor receptor 2 (HER2)-positive tumor received anti-HER2 targeted therapy. Surgical resections were performed 2–3 weeks after completing NAC. The status of estrogen receptor (ER), progesterone receptor (PR), HER2, and the Ki-67 index were determined by immunohistochemistry. We defined tumors with greater than or equal to 1% cells with nuclear staining as ER/PR positive, and the cut-off for Ki-67 was set at 20%. In terms of HER2, tumors with an immunohistochemistry staining score of 0 or 1+ were defined as negative, while those 3+ were defined as positive. The histopathologic diagnosis was independently reviewed by two breast pathologists with 5 and 7 years of experience in breast pathology, respectively. The status of SLNs and ALNs were confirmed by combining the results of SLNB and ALND. During surgery, SLN tracking agents such as blue dye and radioisotope were injected to aid in the identification of SLNs. The false negative rate, was determined by calculating the proportion of patients with negative SLN but positive NSLN, relative to the number of patients who had at least one involved lymph node among those in whom at least one sentinel node was detected^5^. If we calculate the false negative rate of AI-assisted surgery, the false negative rate is defined as the proportion of patients in whom the sentinel lymph node is negative and the AI model also predicts negative for ALN, among patients who are pathologically confirmed to have negative NSLN after ALND.

### MRI acquisition and preprocessing

MRI examinations were conducted using either a 1.5 T or 3.0 T MRI scanner (Philips, 1.5 T Achieva and 3.0 T Ingenia, Philips Healthcare, Best; Siemens) within 2 weeks prior to and after NAC. The imaging sequences comprised axial nonenhanced T1-weighted imaging (T1WI), T2-weighted imaging (T2-WI) with fat suppression, dynamic contrast-enhanced imaging (DCE), and diffusion-weighted imaging (DWI). The DCE sequences were collected after an initial fat-saturated T1-weighted image. Specifically, DCE images during the 90–120 s after contrast agent injection were collected. At this period, the signal intensity of tumor tissue and normal breast tissue exhibit a pronounced contrast, facilitating the accurate delineation of the tumor area. DWI was acquired with a b value of 0 to ensure uniformity across different institutions. Following the intravenous injection of 0.2 ml/kg gadolinium contrast agent (Magnevist; Bayer Healthcare) within 2 min, postcontrast scan was obtained, with five or more subsequent postcontrast images acquired. Axial DWI images were obtained using two b values (0 and 800 or 1000 s/mm^2^).

Imaging normalization was performed to minimize variations between MRI acquisition protocols. To improve the image reproducibility, the pixel values were normalized to an intensity range of 0–1000, and the voxel size was resampled to 1 mm×1 mm×1 mm.

### Segmentation of tumor and lymph node regions

Two experienced radiologists (reader 1 and reader 2) were responsible for the manual delineation of the tumor region, the 5 mm peritumoral region, and the axillary lymph node region on multiparametric MRI images. The delineation process was carried out using 3D Slicer software (version 4.10.2, www. Slicer.org). To ensure accuracy, certain areas such as the skin, ribs, air, and other irrelevant regions were excluded. Regarding the delineation on different sequences, the delineated regions on the DCE sequence was aligned with the corresponding areas on the other two sequences, T2-WI and DWI.

The delineated lymph node region encompassed all lymph nodes to capture features that fully reflected the characteristics of the ALN. In cases where the lesion was not visible after NAC, a 1 cm×1 cm tumor region was delineated at the original tumor location prior to NAC. This delineated region was considered as the lesion after NAC. The delineation of three regions of interest, namely the tumor, peritumoral, and lymph node regions, was performed based on the DCE, T2, and DWI sequences prior to and after NAC.

### Radiomic feature extraction

Radiomics features were extracted from both original and filtered images using PyRadiomics (V3.0.1, https://github.com/Radiomics/pyradiomics). The filtered images were generated using Laplacian of Gaussian and wavelet filters. The Laplacian of Gaussian filters had kernel sizes of 1, 2, 3, and 4, while the wavelet filter consisted of eight decompositions per level in each of the three dimensions. A total of 1223 features were extracted from each region of interest, resulting in 33 021 features per patient. Finally, we obtained a total of 18 sets of features, comprising nine feature sets before NAC and nine feature sets after NAC. The radiomics features encompassed various categories, including the first order (intensity-based histogram), shape-based, gray level cooccurrence matrix (GLCM), gray level size zone matrix (GLSZM), gray level run length matrix (GLRLM), neighboring gray tone difference matrix (NGTDM) and gray level dependence matrix (GLDM).

The delta-radiomics feature (DRF) of a radiomic feature was computed as the relative net change from its value at the pre-NAC timepoint to its value at the post-NAC timepoint. The DRF was calculated by the formula of F_*delta*_=(F_*pre*_ - F_*post*_)/F_*pre*_, where F_*delta*_ represents the relative change in the radiomics feature, F_*pre*_ is the pre-NAC feature, and F_*post*_ is the post-NAC feature. After eliminating duplicate features, such as volume size and strength range, a total of 33 021 features were obtained per patient. This comprised 11 007 features before NAC, 11 007 features after NAC, and 11 007 features for the delta-NAC.

### Feature selection

Due to the tumor heterogeneity among the three molecular subtypes of breast cancer, the treatment response to NAC shows a significant difference. For patients with breast cancer, the most significant radiomics features of each molecular subtype were used to construct an independent radiomics model. To obtain the most representative features of each molecular subtype, feature selection was carried out for each subtype as follows: (i) The Mann–Whitney *U* test was used to choose the features correlated with ALN response to NAC. (ii) Spearman correlation analysis was then used to assess the correlation between the selected features. Features that had a correlation coefficient greater than 0.9 and were correlated with the highest number of other features were iteratively eliminated. (iii) The least absolute shrinkage and selection operator (LASSO) regression with 10-fold cross-validation was then used to select the features with nonzero coefficients.

### Model construction and evaluation

The model construction included two parts: the first part was to predict axillary lymph node response to NAC for patients with clinically positive-node, and the second part was to predict NSLN metastasis for patients with intraoperative negative SLN. In the first part, the primary cohort was used for model construction. The external validation cohorts and the prospective cohort were used for the evaluation of the model. We further analyzed the performance of each single-modality model (pre-NAC, post-NAC, and delta-NAC radiomics) and the stacking model integrating them. The base machine learning models, consisting of logistic regression (LR), random forest (RF), and support vector machine (SVM), were constructed. A stacking strategy, SVM with a radial basis function, was applied to combine the outputs of the base models and give a secondary output. Bootstrap method was applied to train the machine learning model. In each repeat training iteration, the sub-training dataset (80% of patients) was used to develop the model, and the sub-validation dataset (20% of patients) was used to assess hyperparameter tuning using the default parameters of the GridSearchCV Function (Python module scikit-learn 0.20). In the second part, the stacking model output of each patient was used as radiomics signature. Considering some other clinicopathological factors may be critical for identifying NSLN metastasis, the univariate and multivariate logistic regression models were performed to integrate the radiomics signature and significant factors, to construct a combining model. A nomogram was drawn to illustrate the feature importance coefficient of each selected factor.

### Statistical analysis

To compare the differences between groups, Student’s t test or the Mann–Whitney *U* test was used for continuous variables, and the *χ* ^2^ test or Fisher’s exact test was used for categorical variables. Performance was assessed by the area under the receiver operating characteristic curve (AUC) with 95% CI. The sensitivities, specificities, positive predictive values, and negative predictive values were then calculated in the primary and validation cohorts. The optimal cut-off of the radiomics score was determined by maximizing the Youden index in the primary cohort. Then, those fixed cut-off values were applied to the test cohort. The DeLong test was used to compare the AUCs among the radiomics scores. All statistical tests were two-sided, and *P* values <0.05 indicated statistical significance. All statistical analyses were performed using R software (version 3.5.0).

## Results

### Baseline characteristics of patients

**Figure 1:**
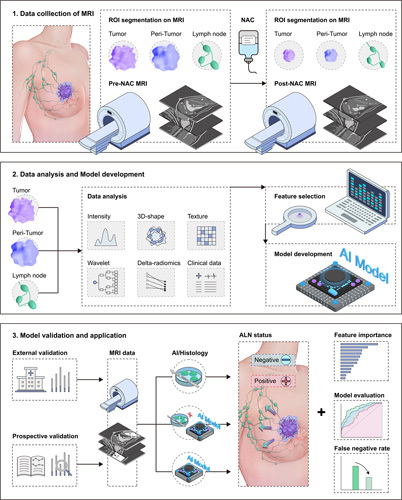
The flowchart of this study. This was a multicenter study with patients enrolled from four institutions. Pre-NAC and post-NAC multiparametric MRI and baseline data were collected. The data from primary cohort was used for model development, and data from the other three cohorts were used as independent validation cohorts.

Table 1 presents the baseline data of all patients, showing no significant differences in clinical characteristics among the cohorts. Figure 1 illustrates the detailed workflow of the study. A total of 461 patients were excluded due to reasons such as the absence of ALND (*n*=127), insufficient MRI data (*n*=265), or inadequate image quality (*n*=69). Eventually, 1038 patients from four institutions were included in the study, consisting of 234 patients from the primary cohort, 723 patients from external validation cohorts, and 81 patients from two prospective clinical trials. Table 1 presents the baseline data of all patients, showing no significant differences in clinical characteristics among the cohorts. The median age of all the patients was 50 years (range: 22–79 years). 80.8% (839/1038) of the patients were diagnosed with clinical stage II or III breast cancer. The average interval between two MRI examinations was 173.5 days, ranging from 132 to 201 days. Overall, 57.71% (599/1038) of the patients experienced a conversion from clinically positive ALN to pathologically negative ALN, which was determined through SLNB and ALND after NAC. Among the patients, 669 patients were confirmed with negative SLN, and 369 had positive SLN. The FNR of SLNB for all patients was 16.31% (70/429). Specifically, when only 1–2 SLNs were removed, the FNR was 25.0% (46/184), whereas it decreased to 9.41% (24/255) when greater than or equal to 3 SLNs were removed.

**Table 1:** Clinical characteristics of patients in the primary and validation cohorts.

Characteristic	Primary cohort (*N*=234)	Validation cohort 1 (*N*=281)	Validation cohort 2 (*N*=151)	Validation cohort 3 (*N*=291)	Prospective cohort (*N*=81)
Age (median, range)	49 (25–71)	49 (25–74)	50 (25–72)	50 (20–79)	52 (22–71)
Clinical T stage (%)					
I	27 (11.54)	29 (10.32)	6 (3.97)	14 (4.81)	3 (3.70)
II	145 (61.97)	175 (62.28)	50 (33.11)	175 (60.14)	52 (64.20)
III	51 (21.79)	33 (11.74)	57 (37.75)	77 (26.46)	24 (29.62)
IV	11 (4.70)	44 (15.66)	38 (25.17)	25 (8.59)	2 (2.47)
Breast surgery (%)					
BCS	63 (26.92)	66 (23.49)	32 (21.19)	76 (26.12)	19 (23.46)
Mastectomy	171 (73.08)	215 (76.51)	119 (78.81)	215 (73.88)	62 (76.54)
HR status (%)					
Positive	155 (66.24)	167 (59.43)	85 (56.29)	177 (60.82)	27 (33.33)
Negative	79 (33.76)	114 (40.57)	66 (43.71)	114 (39.18)	54 (66.67)
HER2 status (%)					
Positive	92 (39.32)	127 (45.20)	75 (49.67)	128 (43.99)	51 (62.96)
Negative	142 (60.68)	154 (54.80)	76 (50.33)	163 (56.01)	30 (37.04)
Ki-67 status (%)					
Positive	217 (92.74)	251 (89.32)	140 (92.72)	262 (90.03)	77 (95.06)
Negative	17 (7.26)	30 (10.68)	11 (7.28)	29 (9.97)	4 (4.94)
SLN status (%)					
Positive	103 (44.02)	80 (28.47)	64 (42.38)	103 (35.40)	19 (23.46)
Negative	131 (55.98)	201 (71.53)	87 (57.62)	188 (64.60)	62 (76.54)
Number of SLN (%)					
1-2	98 (41.88)	113 (40.21)	51 (33.77)	127 (43.64)	26 (32.10)
≥3	136 (58.12)	168 (59.79)	100 (66.23)	164 (56.36)	55 (67.90)

BCS, breast conservation surgery; HER2, human epidermal growth factor receptor 2; HR, hormone receptor; IDC, invasive ductal carcinoma; ILC, invasive lobular carcinoma; SLN, sentinel lymph node.

### Performance of radiomics models

**Figure 2:**
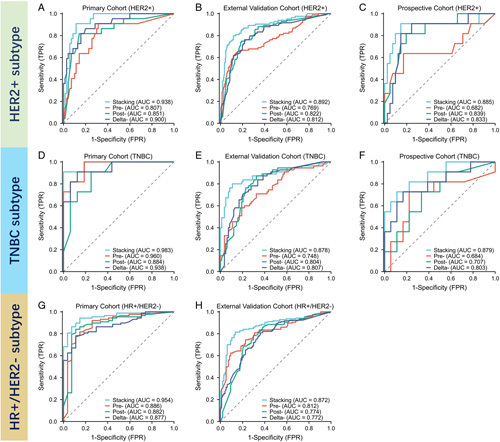
(A–C) The ROC curves among different models and different cohorts in identifying ALN metastasis in HER2+ subtype breast cancer; (D–F) the ROC curves among different models and different cohorts in identifying ALN metastasis in HR+/HER2− subtype breast cancer; (G–I) the ROC curves among different models and different cohorts in identifying ALN metastasis in TNBC subtype breast cancer.

In the first part, we developed radiomics models to predict ALN response to NAC. After feature selection performed in each molecular subtype and dataset, 11 features (3 pre-, 3 post- and 5 delta-) were selected for HR+/HER2- breast cancer, 10 features (3 pre-, 4 post- and 3 delta-) were selected for HR+/HER2- breast cancer, 9 features (3 pre-, 3 post- and 3 delta-) were selected for HR+/HER2- breast cancer. Those features were further used to develop distinct single-scale models and ensemble stacking models for each molecular subtype. The performance of each single-modality model (pre-NAC, post-NAC, and delta-NAC) and their combination by a stacking method are shown in Figure 2. Stacking model achieved superior performance compared to single-scale models. For the HR+/HER2- subtype, the stacking model achieved an AUC of 0.954 (premodel: 0.886; postmodel: 0.882; deltamodel: 0.877) in the primary cohort and an AUC of 0.872 (premodel: 0.812; postmodel: 0.774; deltamodel: 0.772) in the prospective cohort. For the HER2+ subtype, the stacking model performed well with an AUC of 0.938 (premodel: 0.807; postmodel: 0.851; deltamodel: 0.900) in the primary cohort, an AUC of 0.892 (premodel: 0.769; postmodel: 0.822; deltamodel: 0.812) in the external validation cohorts and an AUC of 0.885 (premodel: 0.682; postmodel: 0.839; deltamodel: 0.833) in the prospective cohort. For the TNBC subtype, the stacking model also showed outstanding performance with an AUC of 0.983 (premodel: 0.960; postmodel: 0.884; deltamodel: 0.938) in the primary cohort, an AUC of 0.878 (premodel: 0.748; postmodel: 0.804; deltamodel: 0.807) in the external validation cohorts and an AUC of 0.879 (premodel: 0.684; postmodel: 0.707; deltamodel: 0.803) in the prospective cohort. Thus, the stacking model’s prediction output was used as radiomics signature for each molecular subtype.

The accuracies of stacking model in predicting ALN metastasis were 92.17% (HR+/HER2-), 90.20% (HER2+), and 96.30% (TNBC) in the primary cohort, 83.46% (HR+/HER2-), 88.22% (HER2+), and 85.51% (TNBC) in the external validation cohorts, 88.46 (HER2+) and 82.76% (TNBC) in the prospective validation cohort. And the stacking model performed well in predicting ALN- cases after NAC with specificities of 85.19 (HR+/HER2-), 90.00 (HER2+), and 100.0% (TNBC) in the primary cohort, 86.60 (HR+/HER2-), 93.93 (HER2+), and 89.16% (TNBC) in the external validation cohorts, 90.24 (HER2+) and 88.89% (TNBC) in the prospective validation cohort. The DeLong test also showed that the stacking model significantly improved the model performance for predicting ALN status compared with the three single-modality models (all *P*<0.05) in the external validation cohorts and prospective validation cohort. In all cohorts for each subtype, the decision curve analysis showed that using the stacking model to predict ALN metastasis added more net benefit in a wide range of prediction threshold, which indicated its potential clinical usefulness. The stacking model had higher net benefits than the pre-NAC, post-NAC, and delta-NAC single-scale models in all cohorts. The performance of different radiomics models were shown in Table 2.

**Table 2:** Performances of ensemble radiomics models for predicting axillary lymph node metastasis after NAC in three specific molecular subtypes and different cohorts.

Molecular subtype	Cohort	AUC (95% CI)	ACC (%)	SEN (%)	SPE (%)	PPV (%)	NPV (%)
TNBC	PC	0.983 (0.842–1.0)	96.30	90.91	100.00	100.00	94.12
	EVC	0.878 (0.811–0.927)	85.51	80.00	89.16	83.02	87.06
	PVC	0.879 (0.704–0.970)	82.76	72.73	88.89	80.00	84.21
HER2+	PC	0.938 (0.867–0.977)	90.20	90.91	90.00	71.43	97.30
	EVC	0.892 (0.854–0.924)	88.22	71.43	93.93	80.00	90.63
	PVC	0.885 (0.766–0.957)	88.46	81.82	90.24	69.23	94.87
HR+/HER2−	PC	0.954 (0.898–0.984)	92.17	94.32	85.19	95.40	82.14
	EVC	0.872 (0.825–0.911)	83.46	81.53	86.60	90.78	74.34

AUC, area under the curve; EVC, external validation cohort; NPV, negative predictive value; PC, primary cohort; PPV, positive predictive value; PVC, prospective validation cohort; Sen, sensitivity; SPE, specificity.

### Performance of artificial intelligence-assisted surgery

**Figure 3:**
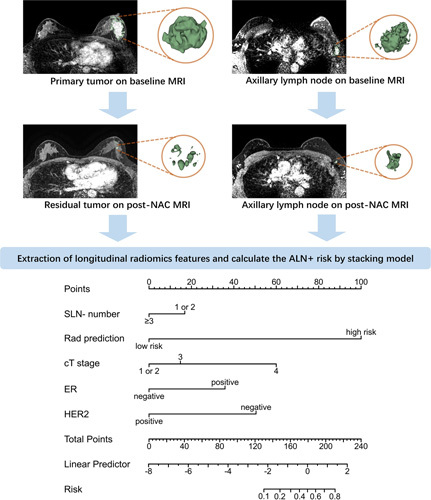
The artificial intelligence-assisted surgery pipeline of this study, and the significant clinical and surgical factors were integrated into the multifactor artificial intelligence model.

**Figure 4:**
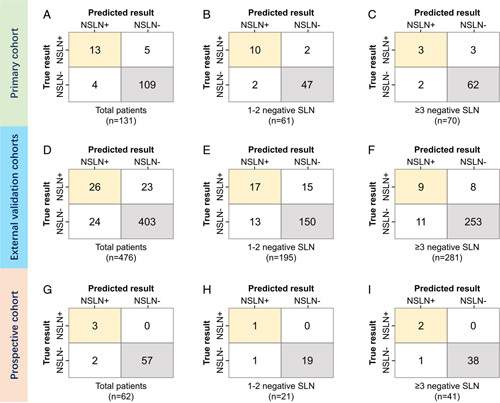
The confusion matrix of true nonsentinel lymph node results and multifactor artificial intelligence model prediction among different cohorts and different sub-groups according the removed sentinel lymph node number. (A–C) results of primary cohort; (D–F) results of external validation cohorts; (G–I) results of prospective validation cohort.

In this study, the average number of SLN removed was 2.9, ranging from 1 to 8 in all patients. In the second part, we investigate the independent predictors of NSLN metastasis in patients with negative SLN, and the age, clinical T stage, clinical N stage, ER, PR, HER2, Ki-67, negative SLN number, and radiomics signature were included. According to the univariate and multivariate logistic regression analysis, the SLN number, cT stage, ER, HER2, and radiomics signature were critical factors to identify NSLN metastasis. For patients in the primary cohort, external validation cohorts, and prospective validation cohort, the FNR of SLNB alone was 24.49, 25.0, and 28.57% when 1–2 SLNs were removed, and 8.33, 10.12, and 6.67% when greater than or equal to 3 SLNs were removed. As shown in the Table 3, the ACCs for predicting NSLN status using SLN number (1 or 2 and more than 3) were 58.02, 62.19, and 67.74% in the primary cohort, external validation cohorts, and prospective validation cohort, respectively. When combining all the significant clinical and surgical factors to develop a clinical model, including SLN number, cT stage, ER and HER2, the ACCs of clinical model were 77.10, 67.02, and 85.48% in the primary cohort, external validation cohorts, and prospective validation cohort, respectively (Figs. 3, 4).

**Table 3:** Performances of the different approach in breast cancer patients with intraoperatively negative sentinel lymph node in different cohorts.

Cohort	Method	ACC (%)	SEN (%)	SPE (%)	PPV (%)	NPV (%)
PC	SLN number	58.02	56.64	66.67	91.43	19.67
	Clinical model	77.10	66.67	78.76	33.33	93.68
	Combining model	93.13	72.22	96.46	76.47	95.61
EVC	SLN number	62.19	61.83	65.31	93.95	16.41
	Clinical model	67.02	46.94	69.32	14.94	91.93
	Combining model	90.13	53.06	94.38	52.00	94.60
PVC	SLN number	67.74	67.80	66.67	97.56	9.52
	Clinical model	85.48	33.33	88.14	12.50	96.30
	Combining model	96.77	100.00	96.61	60.00	100.00

EVC, external validation cohort; NPV, negative predictive value; PC, primary cohort; PPV, positive predictive value; PVC, prospective validation cohort; Sen, sensitivity; SPE, specificity.

**Figure 5:**
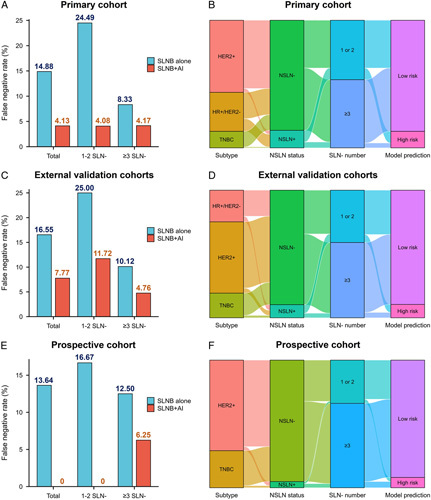
(A, C, E) The false negative rates of sentinel lymph node alone and artificial intelligence-assisted surgery among different cohorts and different sub-groups according the removed sentinel lymph node number. (B, D, F) Sankey diagrams of patients’ axillary lymph node response to neoadjuvant chemotherapy among different cohorts.

When combining all factors to develop a multimodality model, the AI-assisted surgery model further improved the performance with the ACCs of 93.13, 90.13, and 96.77% in the primary cohort, external validation cohorts, and prospective validation cohort, respectively. When 1–2 SLNs were removed, the AI-assisted surgery model significantly reduced the FNR compared with SLNB alone (from 24.49 to 4.08% in the primary cohort, from 25.0 to 11.72% in the external validation cohorts, and from 16.67 to 0% in the prospective validation cohort). Removal of greater than or equal to 3 SLNs has been suggested as a strategy to reduce the FNR in clinical practice, and 60.02% (623/1038) of the overall patients had greater than or equal to 3 SLNs removed. When more than three SLNs were removed, the AI-assisted surgery model further improved the accuracy compared with the SLNB alone, and the FNR was further reduced to 4.17% in the primary cohort, 4.76% in the external validation cohorts, and 6.05% in the prospective validation cohort. Figure 5 illustrated the FNR in different cohorts, which showed that FNR reduced using an artificial intelligence (AI)-based surgery strategy, and the Sankey diagrams showed that the patients’ outcome by the model (Supplemental Digital Content 2, http://links.lww.com/JS9/B184).

## Discussion

In this multicenter study, we developed and validated a longitudinal stacking model integrating multiple spatial radiomics (breast tumor, peritumoral region, and axillary region) and multiple timepoint radiomics (pre-NAC, post-NAC) on MRI images to evaluate ALN response to NAC. More importantly, our study developed an AI-assisted surgery strategy that further improved the accuracy of the model compared with SLNB alone, which indicated the AI-assisted surgery strategy can serve as a potential efficient tool to help axillary surgical decision in breast cancer after NAC.

MRI is a widely used technique for assessing treatment responses to NAC in breast cancer. However, the accuracy of MRI in detecting ALN metastasis after NAC, as assessed by radiologists, varies greatly in terms of sensitivity (ranging from 61% to 85.7%) and specificity (ranging from 54% to 89%)^25–29^. On the other hand, radiomics outperformed human performance in predicting ALN status. Previous studies have attempted to develop pretreatment models using MRI radiomic to assess ALN status after NAC^16,22,26,30^. However, several limitations in these studies have restricted their clinical application. Firstly, their small sample sizes from a single institution reduce the reliability of the radiomics models. Secondly, many studies focused solely on evaluating treatment response based on pre-NAC images and did not achieve optimal performance^15,31^. To address these limitations, longitudinal radiomics enables the evaluation of temporal dynamics change during NAC and can minimize biases associated with different treatment. Recent studies have revealed DRFs could reflect the therapy-induced changes, and improve the performance of the radiomics model^32^. Furthermore, a recent study indicated that the performance of the radiomics model for predicting ALN metastasis based on the tumor and ALN region is better than that of only the tumor or ALN region alone^22^. ALN metastasis depends on the synergistic effect between the primary tumor (seed) and the ALN microenvironment (soil), suggesting that MRI images of the ALN might directly reflect the tumor microenvironment. Therefore, our longitudinal radiomic analysis leveraged MRI images from the tumor, peritumor, and ALN region, along with multiple timepoint analyses (pre-NAC and post-NAC), for assessing ALN status after NAC. This study involved 1038 patients and showed excellent performance in predicting ALN metastasis with an AUC of 0.958 in the primary cohort, and 0.881 in the external validation cohorts. The stacking model was further validated in a prospective validation cohort with an AUC of 0.882, sustaining the use of the longitudinal radiomics model in evaluating nodal response after NAC. The robustness of the longitudinal radiomics model were also excellent, as the AUC deviation of single-modality radiomics model was relatively low by the bootstrap method in the primary cohort, and the AUC deviation was also low in the validation cohorts by repeated validation. That suggested us the imaging-derived feature could be robust by standard and strict feature selection process.

Accurate preoperative assessment of ALN status is crucial to the axillary surgical decision after NAC, sparing some patients from unnecessary ALND. Clinicians often resort to invasive techniques such as double staining, the removal of more than three SLNs, and the placement of positioning needles to reduce the FNR of SLNB to a clinically acceptable level (<10%) in cN+ breast cancer patients following NAC^4–7^. However, the implementation of these techniques presents challenges. The use of radioisotopes in the double staining method is restricted in certain countries due to complex regulations and the difficulties associated with managing radioactive substances. Moreover, in the Z1071 and SENTINA trials, only 56% and 34% of patients, respectively, had three or more SLNs removed^4,5^. Additionally, in 23% of patients, the clipped node was not identified, potentially due to the clips falling out during NAC^7^. Consequently, when SLNB fails to meet the criteria of double staining, removing more than three SLNs, and locating the clipped nodes, the reliability may be compromised, leading to the need for ALND. Currently, there is a lack of a complementary approach to enhance the precision of SLNB. Although some radiomic models have been constructed to predict the status of ALN following NAC^16,22,26,30^, to the best of our knowledge, this is the first model that combines imaging-derived features with clinical and surgical factors to assist in clinical decision-making. In this study, among 255 patients who met the requirement of retrieving ≥ 3 negative SLNs and were also identified as negative ALN status by the AI model, only six patients had positive NSLNs confirmed by final pathology. The overall FNR was reduced to only 2.35%, which will further strengthen our confidence in exempting ALND. On the other hand, if only 1-2 negative SLN were removed, and the AI model predicted negative ALN along with other adverse factors associated with ALN metastasis, such as hormone receptor positivity, HER2 negativity, and a large initial tumor (clinical T3-4 stage), the patient may not be exempted from further ALND. The AI-assisted surgery demonstrated exceptional performance with an AUC above 90.13% to predict the ALN status in the external validation cohorts and 96.77% in the prospective validation cohort. Notably, when the number of SLN removed was less than three, the FNR exceeded 20%, which aligns with the findings from several large multicenter clinical trials^4–6^. However, with the incorporation of AI support in SLNB, the FNR of SLNB could be reduced to below 10%, which is clinically acceptable, even if fewer than three SLN were removed. When more than three SLNs were removed, the FNR of the AI-assisted surgery strategy could be further minimized. These results indicate that the implementation of multifactor AI has the potential to enhance the accuracy of SLNB, even in cases where it does not fulfill the requirements of current invasive techniques, consequently averting unnecessary ALND.

This study represents the largest multicenter investigation of an AI model for predicting ALN status following NAC for breast cancer. The longitudinal stacking radiomics model achieved remarkable performance, with AUCs of 0.958 in the primary cohort, 0.881 in the external validation cohorts, and 0.882 in the prospective validation cohort. Notably, this study is the first research to employ a longitudinal stacking radiomics model combined with clinical and surgical factors to create an integrated learning model for NSLN metastasis prediction. These findings suggest that the integrated multifactor tool may serve as a useful complement to invasive approaches, particularly when SLNB does not meet the criteria for removing more than three nodes.

Our study has some limitations. Firstly, the number of patients in the prospective validation cohort (n=81) was relatively small compared to the large number of enrolled patients (n=1038). A further large-scale prospective cohort including three subtypes of breast cancer is needed to validate this model in the future. Secondly, there was heterogeneity of MRI across centers, although each image had been normalized to reduce heterogeneity and the model achieved excellent performance, the robustness of longitudinal radiomics need to be validated in a larger cohort of breast cancer patients. Thirdly, in addition to MRI, other medical images, such as ultrasound and mammography, are used to evaluate ALN status in clinical practice. The combination of more images may further improve the model.

## Conclusion

We constructed a longitudinal stacking radiomics model based on multiple spatial and multiple timepoint radiomics analyses to identify the ALN metastasis after NAC. In addition, the AI-assisted surgery strategy could further improve the accuracy of SLNB compared with SLNB alone, regardless of the number of SLN removed.

## Ethics statement

Ethical approval for this study (Ethics Approval Number: 20190426) was approved by the Ethics Review Board of the Guangdong Provincial People’s Hospital, Guangzhou, China on 26 April 2019.

## Consent

The requirement for patient approval or written informed consent was waived due to the retrospective nature of this study.

## Sources of funding

This study was supported by grants from the National Natural Science Foundation of China (82171898 and 82103093), Guangdong Basic and Applied Basic Research Foundation (grant number 2020A1515010346, 2021A1515011570, 2022A1515012277), High-level Hospital Construction Project (DFJH202109), Science and Technology Planning Project of Guangzhou City (202002030236 and 202102021055), Beijing Medical Award Foundation (YXJL-2020-0941-0758), and CSCO-Hengrui Cancer Research Fund (Y-HR2016-067). Funding sources were not involved in the study design, data collection, analysis, and interpretation, writing of the report, or decision to submit the article for publication.

## Author contribution

T.Z., Y.-H.H., and W.L.: data curation; T.Z. and Y.-H.H.: formal analysis; K.W.: funding acquisition; Z.-Y.W., G.-L.Y., Y.L., and K.W.: investigation; Y.-H.H. and W.L.: methodology; Z.-Y.W., G.-L.Y., Y.L., and K.W.: project administration; T.Z. and Y.-H.H.: writing – original draft; W.L., Y.-M.Z., Y.-Y.L., and M.-Y.C.: writing – review and editing.

## Conflicts of interest disclosure

The authors declare that they have no financial conflict of interest with regard to the content of this report.

## Research registration unique identifying number (UIN)

NCT03154749 and NCT04858529.

## Guarantor

Kun Wang, MD, PhD, Department of Breast Cancer, Cancer Center, Guangdong Provincial People’s Hospital (Guangdong Academy of Medical Sciences), Southern Medical University, Guangzhou 510080, Guangdong, People’s Republic of China. Tel: +86 13922118086. E-mail: wangkun@gdph.org.cn

## Data availability statement

The datasets used and analyzed during the current study are available from the corresponding author on reasonable request.

## Provenance and peer review

Not commissioned, externally peer-reviewed.

## Supplementary Material
