# Supplementary material for: Multifactor artificial intelligence model assists axillary lymph node surgery in breast cancer after neoadjuvant chemotherapy: multicenter retrospective cohort study
Source: Int J Surg. 2023 Oct 11;109(11):3383–94. doi: 10.1097/JS9.0000000000000621 (PMC10651262; doi:10.1097/JS9.0000000000000621)
Supplement: Supplementary file 2 [file js9-109-3383-s002.docx]

**Supporting Material I: MRI Examination**

The patients which we retrospectively recruited all underwent MRI scan using a 3.0 or 1.5T Tesla system (Siemens Verio, syngo MR B17, Erlangen, Germany) with a dedicated 16-channel breast coil. The image acquisition protocol included fat-suppressed T2WI, fat-suppressed T1+DCE and ADC map images. Following was the detailed description of the data acquisition procedure. At the scanning, an axial fat-suppressed T2WI sequence and axial DWI images were obtained using four b values (0, 800, 1000 and 2000 s/mm2) were acquired before contrast medium administration. An initial fat-saturated T2WI and T1WI pre-contrast scan was collected before T1+DCE images scanning, and then T1+DCE images were acquired as six post contrast scans at intervals of 90 seconds following the intravenous injection of gadolinium contrast agent. A gadolinium-based agent (Magnevist; Bayer Healthcare, Berlin, Germany) was injected using an MR imaging compatible power injector at a rate of 2 ml/s and at a dose of 0.2 ml/kg of body weight, followed by a 20-ml saline flush with high-pressure injector. ADC map was derived from DWI imaging by two b values (the first b value was 0, and the second b value was 1000). Because the second b value was **≥** 800, then we considered the ADC map was not influenced by the b values, and it can reflect the biological information of tumors in imaging accurately.

**Supporting Material II: MRI Imaging Processing and ROI segmentation**

For each MRI sequence, N4ITK MRI bias correction and rotation to volume plane were done. We perform N4ITK MRI bias correction with N4 algorithm in 3D Slicer software (version 4.10.2, [www.slicer.org](http://www.slicer.org)). This algorithm is based on the ITK filters contributed in the following publication: Tustison N, Gee J "N4ITK: Nick's N3 ITK Implementation For MRI Bias Field Correction" (which is introduced in detail at <http://hdl.handle.net/10380/3053>), and the rotation to volume plane was performed using classical module in 3D Slicer software (version 4.10.2, [www.slicer.org](http://www.slicer.org)).

Since the intensity values of MR images distribute widely, we used z-score normalization to make the image intensities have the properties of a standard normal distribution with $\mu=1$ and $\sigma=0$, where $\mu$ is the mean value of the intensities, and $\sigma$ is the standard deviation. The normalized values (also called z scores) of the image intensities (x) were calculated as follows:

$$z= \frac{x-\mu}{\sigma}$$

The segmentation module in the 3D Slicer software (version 4.10.2, [www.slicer.org](http://www.slicer.org)) did the preliminary semi-automatic segmentation according to intensity threshold segmentation. The module is meant to create easy and effecient segmentations on high slice resolution medical images. It can calculate subtraction maps, register images, normalize images, create 3D volumetric ROIs using Delaunay Triangulation, and finally threshold intensities within an ROI. Then the manual corrections such as relabeling and holes filling were done by two professional radiologists with more than ten years of experience in consensus.

Before developing the prediction model, the z score standardization method was used to process the radiomics features in the primary cohort. We first used a feature standard scaler to normalize each radiomics feature by removing the mean and scaling to unit variance. The normalized feature could be calculated as F*_norm_* = (F*_orig_* - F*_m_*)/F*_std_*, where F*_orig_* is the original feature, F*_norm_* is the normalized feature, F*_m_* is the mean value of the feature, and F*_std_* is the standard deviation of the feature. The radiomics features in the validation cohort were transformed according to the F*_m_* and F*_std_* of the feature value in the primary cohort.

After 2 months, 60 patients (30 patients segmented by reader 1 and 30 patients segmented by reader 2) in the primary cohort were randomly selected, and segmentations were performed again by reader 1 and reader 2. After radiomics feature extraction, intraclass correlation coefficients (ICCs) were applied to quantify the feature reproducibility. A feature with an ICC ≥ 0.75, including intra-observer and inter-observer ICC, was considered to have satisfactory reproducibility.

**Supporting Material III: Feature Extraction**

Before features extraction, the voxel size of each sequence was resampled to 1 × 1 × 1 mm3 and the bin width of gray-level histogram was fixed as 25. After z-score normalization of image pixel intensities, a total of 1223 quantitative imaging features including 13 shape based features, 18 first order statistical features, 75 textural features from original images and 1302 derived features (702 features of Gabor-bank wavelet filtered images and 415 features of Law’s filtered images), were extracted respectively for T2 images, DWI and T1-DCE images using corresponding ROIs.

**(1) Shape based features**

In this group of features, we included descriptors of the three-dimensional shape and size of the tumor region. Let in the following definitions V denote the volume and A the surface area of the volume of interest. We determined the following shape and size-based features:

1. **Compactness 1**=$\frac{V}{\sqrt{\pi}A^{\frac{2}{3}}}$
2. **Compactness 2**=$36\pi\frac{V^{2}}{A^{3}}$
3. **Maximum 3d diameter**: The maximum three-dimensional tumor diameter is measured as the largest pairwise Euclidean distance, between voxels on the surface of the tumor volume.
4. **Spherical disproportion** = $\frac{A}{4\pi R^{2}}$
5. **Sphericity** =$\frac{\pi^{\frac{1}{3}}(6V)^{\frac{2}{3}}}{A}$
6. **Surface area**: The surface area is calculated by triangulation (i.e. dividing the surface into connected triangles) and is defined as:

$$A\text{=}\sum_{\text{i}=1}^{N} \frac{1}{2}\left|a_{i}b_{i}\times a_{i}c_{i}\right|$$

Where N is the total number of triangles covering the surface and a, b and c are edge vectors of the triangles.

1. **Surface to volume ratio** =$\frac{A}{V}$
2. **Volume**: The volume (V) of the tumor is determined by counting the number of pixels in the tumor region and multiplying this value by the voxel size.

**(2) First order statistical features**

The following 17 statistical features were extracted.

Let **X** be the three dimensional image matrix with *N* voxels of the ROI and P be the first order histogram distribution with *N_g_* discrete intensity levels.

1. **IntensityMax:** The maximum intensity value of **X.**
2. **IntensityMin:** The minimum intensity value of **X**.
3. **Median:** The median intensity value of **X**.
4. **IntensityStd:**

$$IntensityStd=(\frac{1}{L*W*H-1}\sum_{i=1}^{L} \sum_{j=1}^{W} \sum_{k=1}^{H} (X(\mathrm{i},\mathrm{j},\mathrm{k})-IntensityAve)^{2})^{1/2}$$

1. **Mean:**

$$\frac{1}{N}\sum_{i}^{N} X(i)$$

1. **Variance:**

$$\sqrt{\sum_{j=1}^{N_{g}} P(\mathrm{j})*(\mathrm{j}-\sum_{i=1}^{N_{g}} P(\mathrm{i})*\mathrm{i})^{2}}$$

1. **Skewness:**

$$\sqrt{\sum_{j=1}^{N_{g}} P(\mathrm{j})*(\mathrm{j}-\sum_{i=1}^{N_{g}} P(\mathrm{i})*\mathrm{i})^{3}}$$

1. **Kurtosis:**

$$\sqrt{\sum_{j=1}^{N_{g}} P(\mathrm{j})*(\mathrm{j}-\sum_{i=1}^{N_{g}} P(\mathrm{i})*\mathrm{i})^{4}}$$

1. **Range:**

The range of intensity values of **X**.

1. **Mean absolute deviation:**

The mean of the absolute deviations of all voxel intensities around the mean intensity value

1. **Energy:**

$$\sum_{i}^{N} X(i)^{2}$$

1. **Entropy:**

$$\sum_{i=1}^{N_{g}} P(i)\log_{2}P(i)$$

1. **Entropy_p:**

$$\sum_{i=1}^{N_{g}} \frac{P(i)}{N}\log_{2}\frac{P(i)}{N}$$

1. **Root mean square:**

$$\sqrt{\frac{\sum_{i}^{N}X(i)^{2}}{N}}$$

1. **Uniformity:**

$$\sum_{i=1}^{N_{g}} P(i)^{2}$$

1. **Uniformity_p:**

$$\sum_{i=1}^{N_{g}} (\frac{P(i)}{N})^{2}$$

1. **Mass:**

The sum intensity value of $X$.

**(3) Textural features**

Second order statistic texture features, and higher order statistic texture features were extracted. Forty-four second order statistic texture features could be calculated from the Gray Level Co-occurrence Matrix (GLCM). Forty-six high order statistic texture features were calculated from the Gray Level Size Zone Matrix (GLSZM), Gray Level Run Length Matrix (GLRLM), and Neighborhood Gray Tone Difference Matrix (NGTDM). All of the GLCM, GLSZM, GLRLM, and NGTDM based texture feature were calculated using a 2D analysis and then averaged for all slices within the three-dimensional tumor volume.

*Gray-Level Co-Occurrence Matrix based features (GLCM)*

GLCM based features were second-order statistical texture features, which are defined as a matrix *M* (*i, j; δ, θ*) to indicate the relative frequency with intensity values of pixels (*i* and *j*) at the distance of *δ* in direction *θ*.

Let:

*M*(*i, j*) be the co-occurrence matrix for an arbitrary *δ* and *θ*, set *δ=1 and θ=0 and 45*

*N_g_* be the number of discrete intensity levels in the images, set as 25,

*μ* be the mean of *M*(*i, j*),

$m_{x}(\mathrm{i})=\sum_{j=1}^{N_{g}} M(\mathrm{i},\mathrm{j})$ be the marginal row probabilities,

$m_{y}(\mathrm{i})=\sum_{i=1}^{N_{g}} M(\mathrm{i},\mathrm{j})$ be the marginal column probabilities, and *u_y ,_μ_x,_* be the mean of *m_x_* .and *m_y_*

$HX=-\sum_{i=1}^{N_{g}} m_{x}(i)log(m_{x}(i)$,

$HY=-\sum_{i=1}^{N_{g}} m_{y}(i)log(m_{y}(i)$,

$HXY=-\sum_{i=1}^{N_{g}} \sum_{j=1}^{N_{g}} m(i,j)log(m(i,j))$,

$HXY1=-\sum_{i=1}^{N_{g}} \sum_{j=1}^{N_{g}} m(i,j)log(m_{x}(i)m_{y}(j))$.

$HXY2=-\sum_{i=1}^{N_{g}} \sum_{j=1}^{N_{g}} m_{x}(i)m_{y}(j)log(m_{x}(i)m_{y}(j))$.

1. **Energy:**

$$\sum_{i=1}^{N_{g}} \sum_{j=1}^{N_{g}} [\mathrm{M}(\mathrm{i},\mathrm{j})]^{2}$$

1. **Contrast:**

$$\sum_{i=1}^{N_{g}} \sum_{j=1}^{N_{g}} (\mathrm{i}-\mathrm{j})^{2}*M(\mathrm{i},\mathrm{j})$$

1. **Entropy:**

$$\sum_{i=1}^{N_{g}} \sum_{j=1}^{N_{g}} M(\mathrm{i},\mathrm{j})*\log_{2}M(\mathrm{i},\mathrm{j})$$

1. **Homogeneity 1:**

$$\sum_{i=1}^{N_{g}} \sum_{j=1}^{N_{g}} \frac{M(\mathrm{i},\mathrm{j})}{1+|\mathrm{i}-\mathrm{j}|}$$

1. **Homogeneity 2:**

$$\sum_{i=1}^{N_{g}} \sum_{j=1}^{N_{g}} \frac{M(\mathrm{i},\mathrm{j})}{1+|\mathrm{i}-\mathrm{j}|^{2}}$$

1. **Correlation:**

$$\frac{\sum_{i=1}^{N_{g}} \sum_{j=1}^{N_{g}} ijM(\mathrm{i},\mathrm{j})-\mu_{i}(\mathrm{i})\mu_{j}(\mathrm{j})}{\sigma_{x}(\mathrm{i})\sigma_{y}(\mathrm{j})}$$

1. **Variance:**

$$\sum_{i=1}^{N_{g}} \sum_{j=1}^{N_{g}} (\mathrm{i}-\mu)^{2}M(\mathrm{i},\mathrm{j})$$

1. **Sum Average:**

$$\sum_{i=2}^{2N_{g}} iM_{x+y}(\mathrm{i})$$

1. **Sum Entropy:**

$$-\sum_{i=2}^{2N_{g}} M_{x+y}(\mathrm{i})\log_{2}\left[M_{x+y}(\mathrm{i})\right]$$

1. **Dissimilarity:**

$$\sum_{i=1}^{N_{g}} \sum_{j=1}^{N_{g}} |\mathrm{i}-\mathrm{j}|\mathrm{M}(\mathrm{i},\mathrm{j})$$

1. **Inverse Difference Moment:**

$$\sum_{i=1}^{N_{g}} \sum_{j=1}^{N_{g}} \frac{\mathrm{M}(\mathrm{i},\mathrm{j})}{1+(\frac{|\mathrm{i}-\mathrm{j}|^{2}}{N^{2}})}$$

1. **Autocorrelation:**

$$\sum_{i=1}^{N_{g}} \sum_{j=1}^{N_{g}} ijM(\mathrm{i},\mathrm{j})$$

1. **Cluster Prominence**

$$\sum_{i=1}^{N_{g}} \sum_{j=1}^{N_{g}} \left[i+j-u_{x}-u_{y}\right]^{4}M(\mathrm{i},\mathrm{j})$$

1. **Cluster Shade**

$$\sum_{i=1}^{N_{g}} \sum_{j=1}^{N_{g}} \left[i+j-u_{x}-u_{y}\right]^{3}M(\mathrm{i},\mathrm{j})$$

1. **Cluster Tendency**

$$\sum_{i=1}^{N_{g}} \sum_{j=1}^{N_{g}} \left[i+j-u_{x}-u_{y}\right]^{2}M(\mathrm{i},\mathrm{j})$$

1. **Difference Entropy**

$$-\sum_{i=0}^{N_{g}-1} M_{x-y}(i)\log_{2}\left[M_{x-y}(i)\right]$$

1. **Maximum Probability:**

$$\max\left\{M(i,j)\right\}$$

1. **Sum variance**

$$\sum_{i=2}^{2N_{g}} (i-SE)^{2}M_{x+y}(i)$$

1. **Informational measure of correlation 1 (IMC1):**

$$\frac{HXY-HXY1}{\max\left\{HX-HY\right\}}$$

1. **Informational measure of correlation 2 (IMC2):**

$$\sqrt{1-\text{e}^{-2(HXY2-HXY)}}$$

1. **Inverse Difference Moment Normalized (IDMN):**

$$\sum_{i=1}^{N_{g}} \sum_{j=1}^{N_{g}} \frac{M(i,j)}{1+(\frac{\left|i-j\right|^{2}}{N^{2}})}$$

1. **Inverse Difference Normalized (IDN):**

$$\sum_{i=1}^{N_{g}} \sum_{j=1}^{N_{g}} \frac{M(i,j)}{1+(\frac{\left|i-j\right|}{N^{2}})}$$

*Gray Level Run Length Matrix based features (GLRLM)*

GLRLM based features were high-order statistical texture feature, which were defined as *P*(*i, j; θ*) to indicate the number of times j and gray level i appear consecutively in the direction *θ*.

Let:

*P*(*i, j; θ*) be the run-length matrix *P* for a direction *θ*, set *θ=0 and 45*

*N_g_* be the number of discrete intensity values,

*N_r_* be the number of different run lengths, and

*N_p_* be the number of voxels in the ROI.

1. **Short Run Emphasis (SRE):**

$$\frac{\sum_{i=1}^{N_{g}} \sum_{j=1}^{N_{r}} [\frac{P(\mathrm{i},\mathrm{j};\theta)}{j^{2}}]}{\sum_{i=1}^{N_{g}} \sum_{j=1}^{N_{r}} P(\mathrm{i},\mathrm{j};\theta)}$$

1. **Long Run Emphasis (LRE):**

$$\frac{\sum_{i=1}^{N_{g}} \sum_{j=1}^{N_{r}} j^{2}P(\mathrm{i},\mathrm{j};\theta)}{\sum_{i=1}^{N_{g}} \sum_{j=1}^{N_{r}} P(\mathrm{i},\mathrm{j};\theta)}$$

1. **Gray-Level Nonuniformity (GLN):**

$$\frac{\sum_{i=1}^{N_{g}} [\sum_{j=1}^{N_{r}} P(\mathrm{i},\mathrm{j};\theta)]^{2}}{\sum_{i=1}^{N_{g}} \sum_{j=1}^{N_{r}} P(\mathrm{i},\mathrm{j};\theta)}$$

1. **Run-Length Nonuniformity (RLN):**

$$\frac{\sum_{j=1}^{N_{r}} [\sum_{i=1}^{N_{g}} P(\mathrm{i},\mathrm{j};\theta)]^{2}}{\sum_{i=1}^{N_{g}} \sum_{j=1}^{N_{r}} P(\mathrm{i},\mathrm{j};\theta)}$$

1. **Run Percentage (RP):**

$$\sum_{i=1}^{N_{g}} \sum_{j=1}^{N_{r}} \frac{P(\mathrm{i},\mathrm{j};\theta)}{N_{p}}$$

1. **Low Gray-Level Run Emphasis (LGRE):**

$$\frac{\sum_{i=1}^{N_{g}} \sum_{j=1}^{N_{r}} [\frac{P(\mathrm{i},\mathrm{j};\theta)}{i^{2}}]}{\sum_{i=1}^{N_{g}} \sum_{j=1}^{N_{r}} P(\mathrm{i},\mathrm{j};\theta)}$$

1. **High Gray-Level Run Emphasis (HGRE):**

$$\frac{\sum_{i=1}^{N_{g}} \sum_{j=1}^{N_{r}} i^{2}P(\mathrm{i},\mathrm{j};\theta)}{\sum_{i=1}^{N_{g}} \sum_{j=1}^{N_{r}} P(\mathrm{i},\mathrm{j};\theta)}$$

1. **Short Run Low Gray-Level Emphasis (SRLGE):**

$$\frac{\sum_{i=1}^{N_{g}} \sum_{j=1}^{N_{r}} \frac{P(\mathrm{i},\mathrm{j};\theta)}{i^{2}j^{2}}}{\sum_{i=1}^{N_{g}} \sum_{j=1}^{N_{r}} P(\mathrm{i},\mathrm{j};\theta)}$$

1. **Short Run High Gray-Level Emphasis (SRHGE):**

$$\frac{\sum_{i=1}^{N_{g}} \sum_{j=1}^{N_{r}} \frac{i^{2}P(\mathrm{i},\mathrm{j};\theta)}{j^{2}}}{\sum_{i=1}^{N_{g}} \sum_{j=1}^{N_{r}} P(\mathrm{i},\mathrm{j};\theta)}$$

1. **Long Run Low Gray-Level Emphasis (LRLGE):**

$$\frac{\sum_{i=1}^{N_{g}} \sum_{j=1}^{N_{r}} \frac{j^{2}P(\mathrm{i},\mathrm{j};\theta)}{i^{2}}}{\sum_{i=1}^{N_{g}} \sum_{j=1}^{N_{r}} P(\mathrm{i},\mathrm{j};\theta)}$$

1. **Long Run High Gray-Level Emphasis (LRHGE):**

$$\frac{\sum_{i=1}^{N_{g}} \sum_{j=1}^{N_{r}} i^{2}j^{2}P(\mathrm{i},\mathrm{j};\theta)}{\sum_{i=1}^{N_{g}} \sum_{j=1}^{N_{r}} P(\mathrm{i},\mathrm{j};\theta)}$$

1. **Mean:**

$$\frac{1}{2N_{\text{g}}}\sum_{i=1}^{N_{g}} \sum_{j=1}^{N_{g}} [P(\mathrm{i},\mathrm{j})]^{2}$$

1. **Entropy:**

$$\sum_{i=1}^{N_{g}} \sum_{j=1}^{N_{g}} P(\mathrm{i},\mathrm{j})*\log_{2}P(\mathrm{i},\mathrm{j})$$

1. **Energy:**

$$\sum_{i=1}^{N_{g}} \sum_{j=1}^{N_{g}} [P(\mathrm{i},\mathrm{j})]^{2}$$

*Gray Level Size Zone Matrix based features (GLSZM)*

GLSZM based features were high-order statistical texture features, which were defined as *P*(*i, j*) to indicate the areas of size j and gray level i.

Let:

*P*(*i, j*) be the size zone of matrix *P*,

*N_g_* be the number of discrete intensity values,

*N_r_* be the number of different areas sizes,

*N_p_* be the number of voxels in the ROI.

1. **Small Zone Emphasis (SZE):**

$$\frac{\sum_{i=1}^{N_{g}} \sum_{j=1}^{N_{r}} [\frac{P(\mathrm{i},\mathrm{j})}{j^{2}}]}{\sum_{i=1}^{N_{g}} \sum_{j=1}^{N_{r}} P(\mathrm{i},\mathrm{j})}$$

1. **Large Zone Emphasis (LZE):**

$$\frac{\sum_{i=1}^{N_{g}} \sum_{j=1}^{N_{r}} j^{2}P(\mathrm{i},\mathrm{j})}{\sum_{i=1}^{N_{g}} \sum_{j=1}^{N_{r}} P(\mathrm{i},\mathrm{j})}$$

1. **Gray-Level Nonuniformity (GLN):**

$$\frac{\sum_{i=1}^{N_{g}} [\sum_{j=1}^{N_{r}} P(\mathrm{i},\mathrm{j})]^{2}}{\sum_{i=1}^{N_{g}} \sum_{j=1}^{N_{r}} P(\mathrm{i},\mathrm{j})}$$

1. **Zone-Size Nonuniformity (ZSN):**

$$\frac{\sum_{j=1}^{N_{r}} [\sum_{i=1}^{N_{g}} P(\mathrm{i},\mathrm{j})]^{2}}{\sum_{i=1}^{N_{g}} \sum_{j=1}^{N_{r}} P(\mathrm{i},\mathrm{j})}$$

1. **Zone Percentage (ZP):**

$$\sum_{i=1}^{N_{g}} \sum_{j=1}^{N_{r}} \frac{P(\mathrm{i},\mathrm{j})}{N_{p}}$$

1. **Low Gray-Level Zone Emphasis (LGZE):**

$$\frac{\sum_{i=1}^{N_{g}} \sum_{j=1}^{N_{r}} [\frac{P(\mathrm{i},\mathrm{j})}{i^{2}}]}{\sum_{i=1}^{N_{g}} \sum_{j=1}^{N_{r}} P(\mathrm{i},\mathrm{j})}$$

1. **High Gray-Level Zone Emphasis (HGZE):**

$$\frac{\sum_{i=1}^{N_{g}} \sum_{j=1}^{N_{r}} i^{2}P(\mathrm{i},\mathrm{j})}{\sum_{i=1}^{N_{g}} \sum_{j=1}^{N_{r}} P(\mathrm{i},\mathrm{j})}$$

1. **Small Zone Low Gray-Level Emphasis (SZLGE):**

$$\frac{\sum_{i=1}^{N_{g}} \sum_{j=1}^{N_{r}} \frac{P(\mathrm{i},\mathrm{j})}{i^{2}j^{2}}}{\sum_{i=1}^{N_{g}} \sum_{j=1}^{N_{r}} P(\mathrm{i},\mathrm{j})}$$

1. **Small Zone High Gray-Level Emphasis (SZHGE):**

$$\frac{\sum_{i=1}^{N_{g}} \sum_{j=1}^{N_{r}} \frac{i^{2}P(\mathrm{i},\mathrm{j})}{j^{2}}}{\sum_{i=1}^{N_{g}} \sum_{j=1}^{N_{r}} P(\mathrm{i},\mathrm{j})}$$

1. **Large Zone Low Gray-Level Emphasis (LZLGE):**

$$\frac{\sum_{i=1}^{N_{g}} \sum_{j=1}^{N_{r}} \frac{j^{2}P(\mathrm{i},\mathrm{j})}{i^{2}}}{\sum_{i=1}^{N_{g}} \sum_{j=1}^{N_{r}} P(\mathrm{i},\mathrm{j})}$$

1. **Large Zone High Gray-Level Emphasis (LZHGE):**

$$\frac{\sum_{i=1}^{N_{g}} \sum_{j=1}^{N_{r}} i^{2}j^{2}P(\mathrm{i},\mathrm{j})}{\sum_{i=1}^{N_{g}} \sum_{j=1}^{N_{r}} P(\mathrm{i},\mathrm{j})}$$

1. **Gray-Level Variance (GLV):**

$$\sum_{i=1}^{N_{g}} \sum_{j=1}^{N_{r}} \{\mathrm{iP}(\mathrm{i},\mathrm{j})-\frac{\sum_{i=1}^{N_{g}} i[\sum_{j=1}^{N_{r}} P(\mathrm{i},\mathrm{j})]}{N_{g}N_{r}}\}$$

1. **Zone-Size Variance (ZSV):**

$$\sum_{i=1}^{N_{g}} \sum_{j=1}^{N_{r}} \{\mathrm{jP}(\mathrm{i},\mathrm{j})-\frac{\sum_{i=1}^{N_{g}} i[\sum_{j=1}^{N_{r}} P(\mathrm{i},\mathrm{j})]}{N_{g}N_{r}}\}$$

*Neighborhood Gray Tone Difference Matrix based features (NGTDM)*

NGTDM based features were high-order statistical texture features, which were defined as *S(i)* to indicate the sum of the absolute value between gray intensity level i and it’s neighbors’ average intensity.

Let:

*S(i)* be the sum of absolute value between gray intensity level i and its neighbors’ average intensity,

*C(i)* be the number of voxels with the gray intensity level I,

*N_g_* be the number of discrete intensity values.

1. **Coarseness:**

$$\frac{1}{\varepsilon+\sum_{i=1}^{N_{g}} \frac{C(\mathrm{i})\mathrm{S}(\mathrm{i})}{\sum_{i=1}^{N_{g}} C(\mathrm{i})}}$$

1. **Contrast:**

$$\frac{\sum_{i=1}^{N_{g}} \sum_{j=1}^{N_{g}} C(\mathrm{i})\mathrm{C}(\mathrm{j})(\mathrm{i}-\mathrm{j})^{2}}{(\sum_{i=1}^{N_{g}} C(\mathrm{i}))^{2}}*\sum_{i=1}^{N_{g}} S(\mathrm{i})*\frac{1}{N_{g}(\mathrm{N}_{g}-1)\sum_{i=1}^{N_{g}} C(\mathrm{i})}$$

1. **Busyness:**

$$\frac{\sum_{i=1}^{N_{g}} C(\mathrm{i})\mathrm{S}(\mathrm{i})}{\sum_{i=1}^{N_{g}} \sum_{j=1}^{N_{g}} (\mathrm{iC}(\mathrm{i})-\mathrm{jC}(\mathrm{j}))}, C(\mathrm{i})\neq0,\mathrm{C}(\mathrm{j})\neq0$$

1. **Complexity:**

$$\frac{1}{(\sum_{i=1}^{N_{g}} C(\mathrm{i}))^{2}}*\sum_{i=1}^{N_{g}} \sum_{j=1}^{N_{g}} \frac{|\mathrm{i}-\mathrm{j}|(\mathrm{C}(\mathrm{i})\mathrm{S}(\mathrm{i})+\mathrm{C}(\mathrm{j})\mathrm{S}(\mathrm{j}))}{(\mathrm{C}(\mathrm{i})+\mathrm{C}(\mathrm{j}))}, C(\mathrm{i})\neq0,\mathrm{C}(\mathrm{j})\neq0$$

1. **Strength:**

$$\frac{\sum_{i=1}^{N_{g}} \sum_{j=1}^{N_{g}} (\mathrm{C}(\mathrm{i})-\mathrm{C}(\mathrm{j}))*(\mathrm{i}-\mathrm{j})^{2}}{\sum_{i=1}^{N_{g}} C(\mathrm{i})*\sum_{i=1}^{N_{g}} S(\mathrm{i})}, C(\mathrm{i})\neq0,\mathrm{C}(\mathrm{j})\neq0$$

**(4) Wavelet features: first order statistical and texture features of a wavelet filtered image.**

A total of 1223 derived wavelet features were extracted for each sequence, with the Gaussian filter and a wavelet-based filter. These features were computed on the filtered images. The original image was filtered by 8 filters. For each image, the first order statistical and texture features were computed.

**Supporting Material IV: Feature Selection Algorithm**

**(1) LASSO (least absolute shrinkage and selection operator)**

The [Lasso](https://scikit-learn.org/stable/modules/generated/sklearn.linear_model.Lasso.html#sklearn.linear_model.Lasso) is a linear model that estimates sparse coefficients. It is useful in some contexts due to its tendency to prefer solutions with fewer non-zero coefficients, effectively reducing the number of features upon which the given solution is dependent. For this reason Lasso and its variants are fundamental to the field of compressed sensing. Under certain conditions, it can recover the exact set of non-zero coefficients. As the Lasso regression yields sparse models, it can thus be used to perform feature selection, as detailed in [L1-based feature selection](https://scikit-learn.org/stable/modules/feature_selection.html#l1-feature-selection).

**(2) Boruta**

Boruta algorithm uses a wrapper method based on the RF classifier for feature selection. A “shadow” attribute was created for each feature in the feature pool by shuffling values of the original feature across all patients. Then the shadow attributes are combined with original features for classification using an RF model. The importance of shadow attribute is used as a reference for selecting truly important features, as determined by RF permutation importance measure. In the implementation of Boruta of our study, the number of trees for RF was set to 500 and the maximum number of importance source runs was set to 100.

**Supporting Material V: Machine Learning Algorithms**

**(1) Random Forest and XGboost**

Random forest consists of multiple decision trees. Random forest is a discriminant model that supports both classification and regression problems, and supports multiple classification problems. It is a nonlinear model. For classification problems, a test sample will be sent to each decision tree for prediction, and then voted, the category with the most votes is the final classification result. In addition, when performing node segmentation in the process of building a tree, the selected segmentation point is no longer the best segmentation point among all features, but the optimal segmentation point in a random subset of features. Due to this randomness, the deviation of the forest usually increases slightly (relative to the deviation of a single non-random tree), but because the average is taken, its variance will also decrease, and it can usually compensate for the increase in deviation, resulting in an overall better model. “n_estimators” and “criterion” were used in the tuning step of model development.

**(2) Logistics Regression**

Logistic regression is a linear regression model, which assumes that the data obey Bernoulli distribution. By means of maximum likelihood function, the gradient descent method is used to solve the parameters, so as to achieve the purpose of dichotomy.LR model can be considered as a linear regression model normalized by Sigmoid function (Logistic equation). Sigmoid compresses the data (LR, middle finger, output y) between [0, 1] and passes through an important point (0, 0.5). In this way, the output is compressed between [0,1], with 0.5 as the boundary value, 0.5 greater than 0.5 as one class, and 0.5 less than 0.5 as another class. “C”, “penalty” and “solver” were used in the tuning step of model development.

**(3) Support Vector Machine**

SVM mainly used to solve data classification problems in the field of pattern recognition, which belongs to a kind of supervised learning algorithm. It has strong nonlinear classification ability, showing many unique advantages in solving small sample, nonlinear and high-dimensional pattern recognition problems. “C” and “gamma” were used in the tuning step of model development.

**Supporting Material VI: Machine Learning Algorithms**

Considering that it is unrealistic for our patients to use the same magnetic resonance instrument before and after the NAC in real clinical operation, we have performed some important steps to balance the MRI data, including N4 bias field correction (MR scans often display intensity non-uniformities due to variations in the magnetic field. So, one part of an image might appear lighter or darker when visualized, solely because of variations in the magnetic field. The map of these variations is called the bias field. The bias field can cause problems for a classifier as the variations in signal intensity are not due to any anatomical differences, seeing this part in the supplementary materials), spacing voxel resample (normalizing the voxels of different sizes of medical images to the same size, the resampling spacing are 1mm * 1mm * 1mm) and the gray scale normalization (all the images were normalized to 0-1000, and the bin width is 25). Even for the radiomics features, we repeated the ROI segmentation to calculate the Inter- and intra-class correlation coefficients, and only feature with ICCs >0.75 were retained for further analysis. Performing those steps, we considered that the influence of image parameter inconsistency on radiomics feature variation had been reduced. In order to ensure the robustness of MRI in the two examinations before and after NAC and the robustness between different centers, we also compared the robustness of the radiomics features of patients with the same ALN category in different centers. Among them, the top selected features were robust after combat for each molecular subtype due to no difference was observed of the radiomics feature value among institutions (p > 0.05 by student’s t test). Therefore, we thought that the radiomics features extracted from different magnetic resonance images are stable enough.

 In radiomics analysis of breast cancer, radiomics features are mathematically defined and classified into morphology, histogram, texture, or transformed features. Morphology features are compactness, roundness, or convexity. Histogram features are median, entropy, uniformity, skewness, or kurtosis, in which spatial information is not included. Texture features include the spatial information. Gray-level co-occurrence matrix (GLCM)-based features analyzed the relationship between voxels and their neighborhoods, including entropy, contrast, and homogeneity. Transform-based features such as Laplacian of Gaussian and wavelet are commonly used to transform the original image to create a new image from which the features can be quantified. most radiomics features defined below are in compliance with feature definitions as described by the Imaging Biomarker Standardization Initiative (IBSI). In our study, following the IBSI guidelines, we did the magnetic field correction, the intensity normalization of voxels, the resampling of the images to the same spatial resolution, and the standardization of radiomics features according to the suggestion of relative articles. I thought our study was exploratory research to investigate whether the longitudinal radiomics features could reflect the treatment response of breast cancer to NAC, and the evidences we provided had strong repeatability.

**Supporting Material VII: False negative rate calculation**

The false negative rate, was determined by calculating the proportion of patients with negative sentinel lymph nodes but positive non-sentinel lymph nodes, relative to the number of patients who had at least one involved lymph node among those in whom at least one sentinel node was detected. The calculation formula of FNR for sentinel lymph node biopsy was as follow:

*FNR = the number of patients (SLN-/NSLN+) / the number of patients (ALN+)*

If we calculate the false negative rate of AI-assisted surgery, the false negative rate is defined as the proportion of patients in whom the sentinel lymph node is negative and the artificial intelligence-assisted surgery strategy also predicts negative for non-sentinel lymph node, among patients who are pathologically confirmed to have negative non-sentinel lymph nodes after axillary lymph node dissection. The calculation formula of FNR for artificial intelligence-assisted surgery strategy was as follow:

*FNR = the number of patients (SLN-/NSLN+, and low risk predicted by AI model) / the number of patients (ALN+)*

**sFigure 1.** the segmentation of tumor region and 5-mm peri-tumor region at baseline DCE MRI image, and we only delineated the enhanced region for study. During ROI segmentation, the necrosis, air and calcification area of the breast cancer were excluding carefully


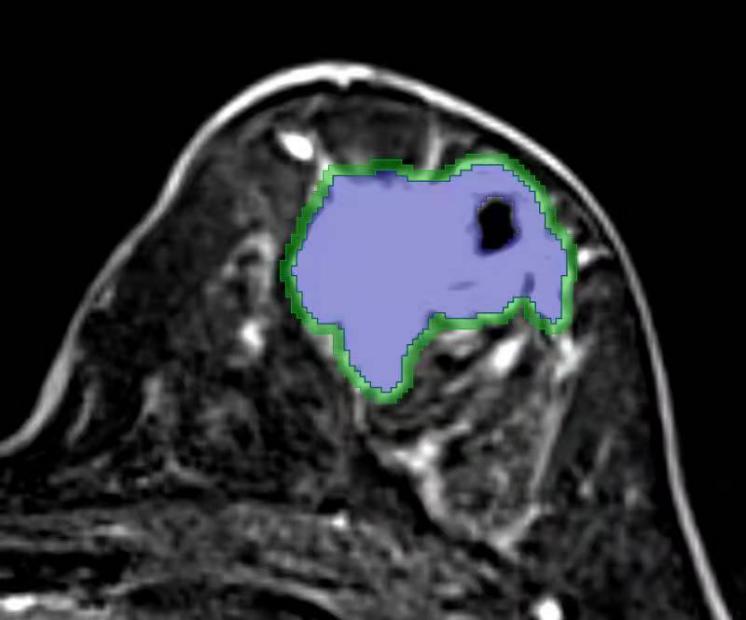


**sFigure 2.** the spearman correlation coefficient among selected radiomics features and clinicopathological factors in HR+/HER2- subtype


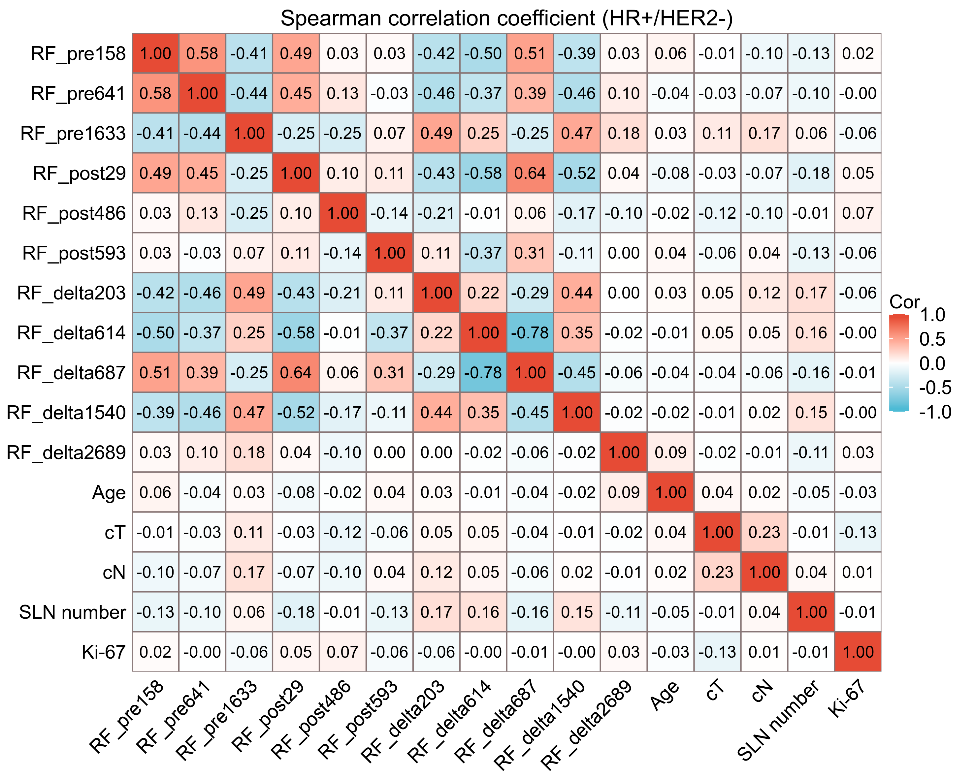


**sFigure 3.** the feature contribution coefficient to machine learning model in HR+/HER2- subtype


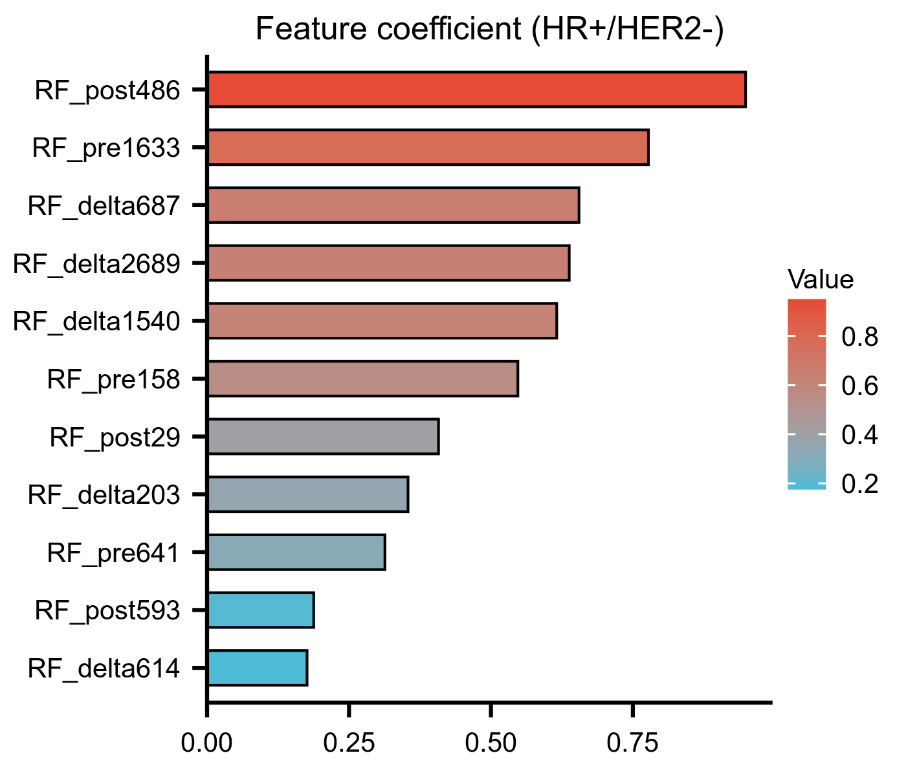


**sFigure 4.** the spearman correlation coefficient among selected radiomics features and clinicopathological factors in HER2+ subtype


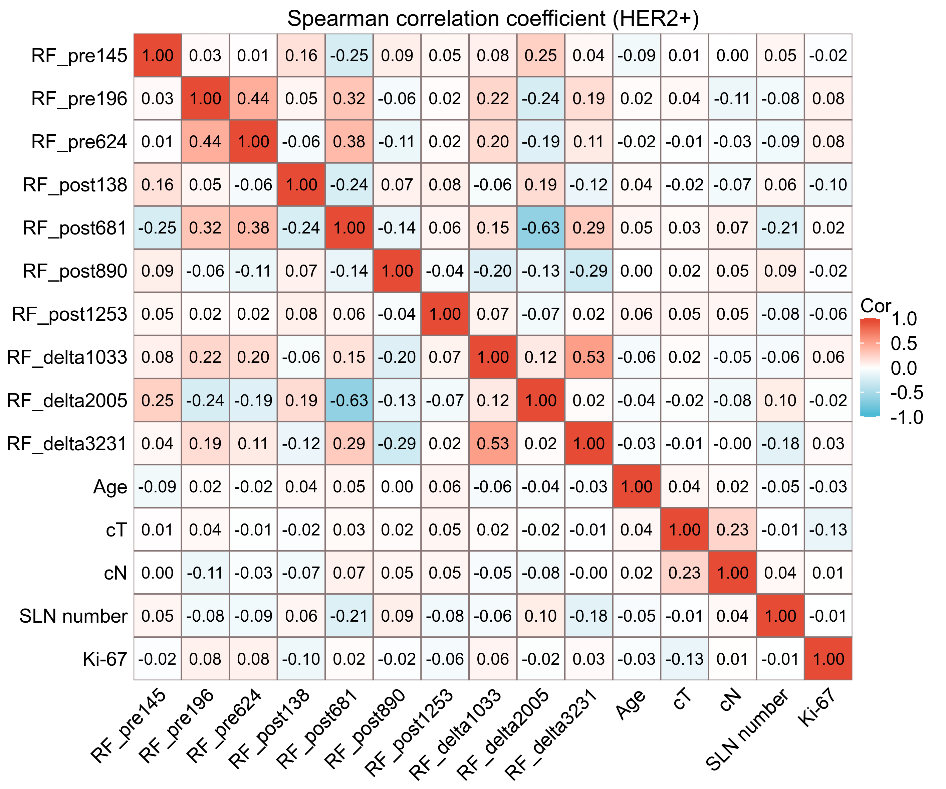


**sFigure 5.** the feature contribution coefficient to machine learning model in HER2+ subtype


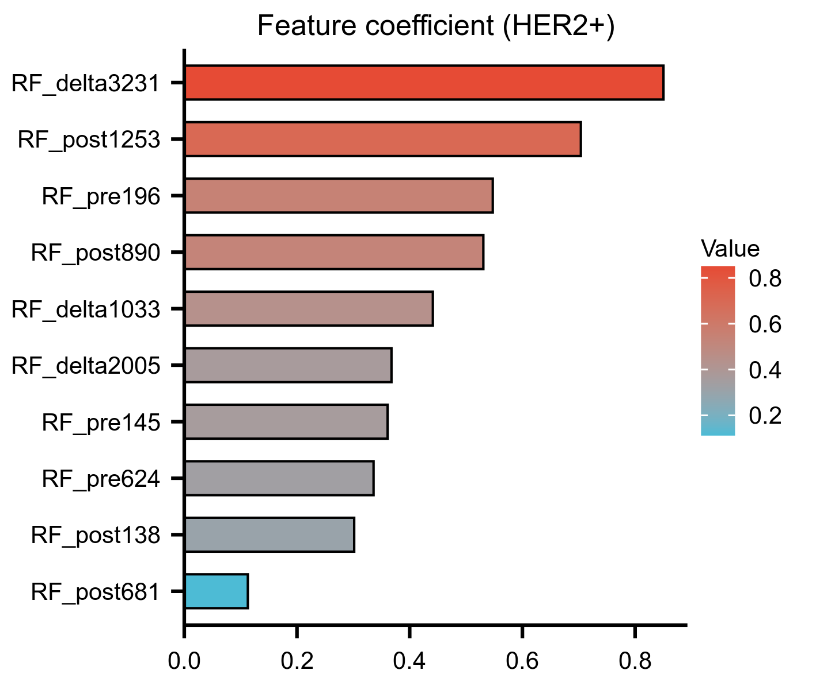


**sFigure 6.** the spearman correlation coefficient among selected radiomics features and clinicopathological factors in TNBC subtype


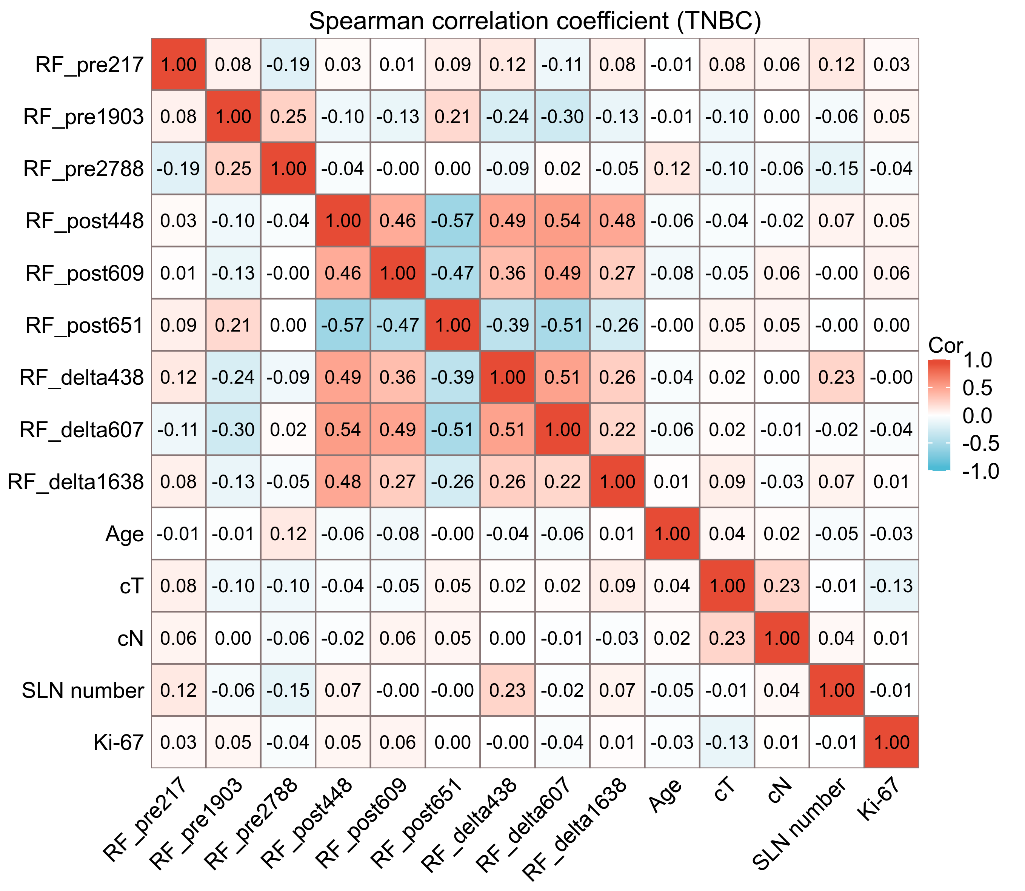


**sFigure 7.** the feature contribution coefficient to machine learning model in TNBC subtype


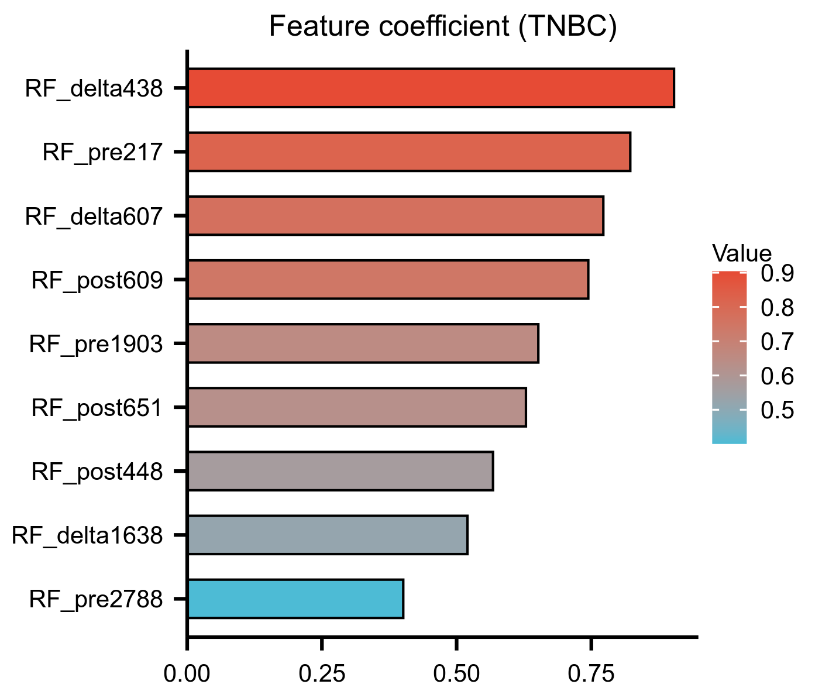


**sFigure 8.** The nomogram of multi-factor artificial intelligence to predict non-sentinel lymph node in breast cancer.


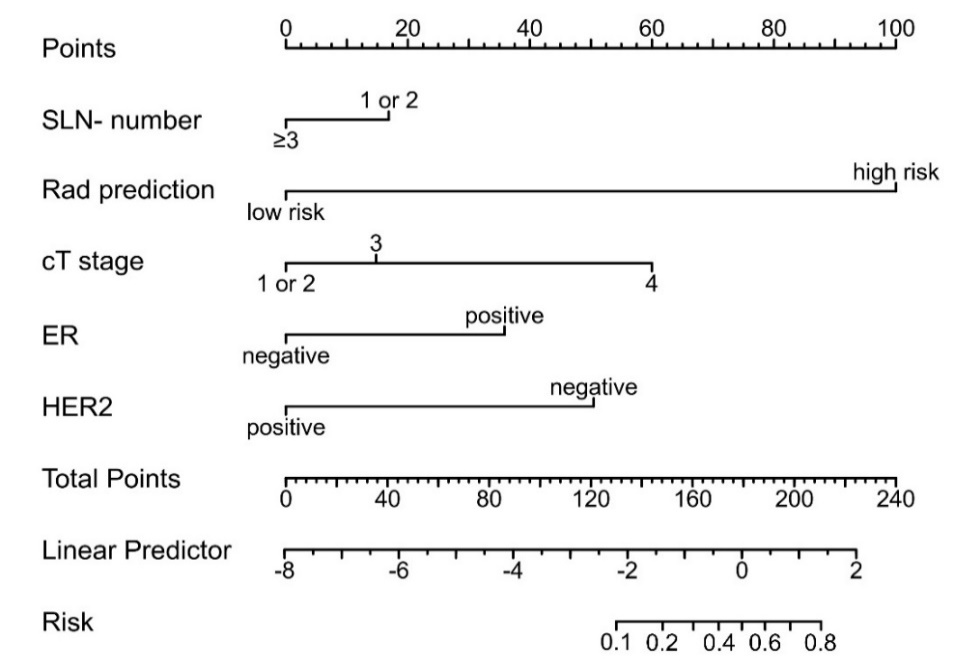


**sFigure 9.** The area under the curve distribution in bootstrap circulation of different cohorts


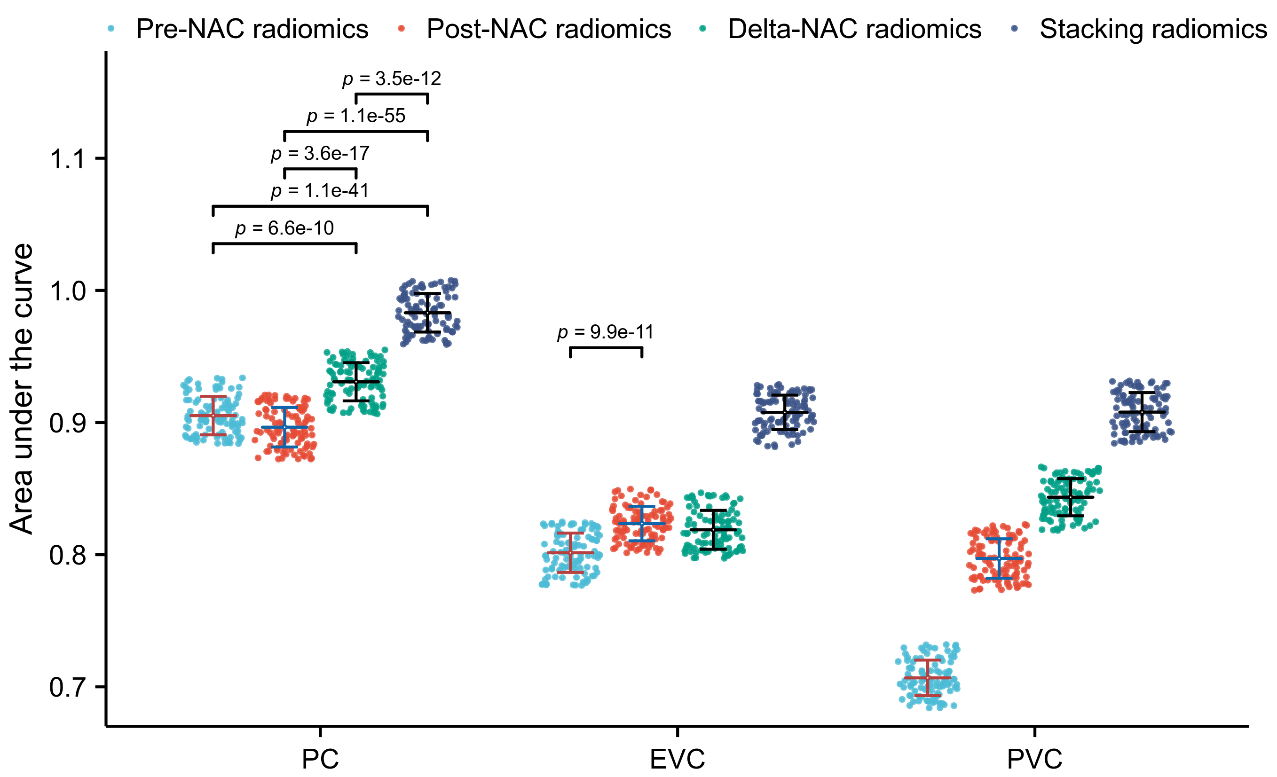


**sTable 1.** MR scanning parameters for the patients in four cohorts

| **Cohorts** | **Scanner** | **Sequence** | **TR/TE (ms)** | **FOV (mm)** | **Matrix** | **Slice Thickness (mm)** | **Slice Gap (mm)** | **Flip Angle** |
| --- | --- | --- | --- | --- | --- | --- | --- | --- |
| PC | Philips 1.5T (Achieva) | T2WI | 3400/90 | 260×320 | 348×299 | 3 | 0.3 | 120° |
|  |  | DWI | 2000/103 | 320×320 | 160×160 | 5 | 1 | 90° |
|  |  | T1+C | 5.4/2.4 | 300×320 | 300×320 | 1 | 0 | 15° |
| VC-1 | Siemens 1.5T (Avanto) | T2WI | 5600/56 | 340×340 | 320×313 | 4 | 0.8 | 142° |
|  |  | DWI | 4900/84 | 185×340 | 220×220 | 4 | 0.8 | 90° |
|  |  | T1+C | 4.43/1.5 | 340×340 | 448×336 | 1.7 | 0.34 | 10° |
| VC-2 | GE 1.5T (Signa HDx) | T2WI | 5950/85 | 320×320 | 320×192 | 4 | 0.5 | 90° |
|  |  | DWI | 8000/86.8 | 320×320 | 128×128 | 4.5 | 0.5 | 90° |
|  |  | T1+C | 6.5/2.1 | 380×342 | 256×256 | 3 | 0 | 10° |
| VC-3 | Siemens 3.0T (Skyra) | T2WI | 3600/54 | 340×319 | 384×384 | 4 | 0.4 | 120° |
|  |  | DWI | 6700/93 | 340×153 | 200×200 | 4 | 0.4 | 180° |
|  |  | T1+C | 5.65/2.46 | 360×360 | 384×384 | 2.5 | 0.5 | 15° |

**sTable 2.** Performances of different models and radiologist’s evaluation for predicting axillary lymph node response in different cohorts

| **Cohort** | **Model** | **Macro AUC** | **ACC (%)** | **SEN (%)** | **SPE (%)** | **PPV (%)** | **NPV (%)** |
| --- | --- | --- | --- | --- | --- | --- | --- |
| PC | Stacking | 0.958 | 91.80 | 93.39 | 90.24 | 90.40 | 93.28 |
|  | Pre | 0.884 | 84.48 | 86.06 | 79.45 | 89.73 | 81.23 |
|  | Post | 0.872 | 87.01 | 89.48 | 81.43 | 90.81 | 88.75 |
|  | Delta | 0.905 | 86.06 | 84.61 | 85.64 | 79.73 | 84.35 |
|  | Tumor change | 0.725 | 68.38 | 45.13 | 90.08 | 80.95 | 63.74 |
|  | ALN change | 0.745 | 73.93 | 90.27 | 58.68 | 67.11 | 86.59 |
| EVC | Stacking | 0.881 | 86.03 | 78.38 | 91.33 | 86.25 | 85.90 |
|  | Pre | 0.776 | 78.94 | 76.30 | 80.41 | 76.82 | 83.63 |
|  | Post | 0.800 | 82.35 | 78.60 | 86.69 | 79.12 | 84.30 |
|  | Delta | 0.797 | 80.41 | 76.71 | 82.44 | 77.67 | 86.29 |
|  | Tumor change | 0.695 | 64.45 | 48.48 | 87.50 | 84.84 | 54.07 |
|  | ALN change | 0.731 | 75.93 | 88.76 | 57.43 | 75.05 | 77.98 |
| PVC | Stacking | 0.882 | 86.42 | 77.27 | 89.83 | 73.91 | 91.38 |
|  | Pre | 0.683 | 72.35 | 62.72 | 81.34 | 65.71 | 80.55 |
|  | Post | 0.773 | 82.94 | 81.72 | 83.04 | 70.00 | 90.95 |
|  | Delta | 0.818 | 79.70 | 72.72 | 85.04 | 67.22 | 88.42 |
|  | Tumor change | 0.683 | 64.20 | 59.32 | 77.27 | 87.50 | 41.46 |
|  | ALN change | 0.702 | 75.31 | 81.36 | 59.09 | 84.21 | 54.17 |

Abbreviation: AUC, the area under curve; ACC, accuracy; Sen, sensitivity; SPE, specificity; PPV, positive predictive value; NPV, negative predictive value; PC, primary cohort; EVC, external validation cohort; PVC, prospective validation cohort
